# Enhancing Muscle Quality: Exploring Leucine and Whey Protein in Sarcopenic Individuals

**DOI:** 10.1002/jcsm.70060

**Published:** 2025-09-12

**Authors:** Aiman Ijaz, Huma Bader Ul Ain, Tabussam Tufail, Raima Mariam, Sana Noreen, Aniqa Amjad, Ali Ikram, Muhammad Tayyab Arshad, Muhammed Adem Abdullahi

**Affiliations:** ^1^ University Institute of Diet and Nutritional Sciences The University of Lahore Lahore Pakistan; ^2^ School of Food Science and Engineering Yangzhou University Yangzhou China; ^3^ University Institute of Food Science and Technology The University of Lahore Lahore Pakistan; ^4^ Functional Food and Nutrition Program, Faculty of Agro‐Industry Prince of Songkla University Songkhla Thailand; ^5^ Department of Food Science and Postharvest Technology, College of Agriculture and Veterinary Medicine Jimma University Ethiopia

**Keywords:** mTOR signalling pathway, muscle health, whey protein

## Abstract

**Background:**

Sarcopenia is characterised by a decline in the skeletal muscle mass with increasing age. This poses many challenges to the health and independence of older individuals. New and emerging studies have focused on supplementing leucine‐enriched whey protein and resistance training in older adults to mitigate the effects of sarcopenia, including significant muscle mass loss.

**Methods:**

This study aimed to comprehensively analyse the current literature regarding the beneficial effects of leucine‐enriched whey protein supplementation in older adults. This article systematically reviewed randomised controlled trials, longitudinal studies, and meta‐analyses published between 2011 and 2024. The biochemical mechanisms underlying the anabolic properties of leucine, including its role in protein synthesis and mTOR signalling pathways, were explored to elucidate its potential to enhance muscle protein accretion.

**Results:**

Findings from the reviewed studies indicate that the combined intervention of leucine‐enriched whey protein supplementation and resistance training elicits favourable outcomes regarding increased lean muscle mass, muscle strength and physical performance in older adults with sarcopenia. The potential of leucine‐enriched whey protein to mitigate muscle protein breakdown is discussed along with its potential synergistic effects with resistance exercise to optimise muscle adaptation. Future research directions, including investigations of optimal dosing regimens, long‐term sustainability and potential contraindications, are proposed to enhance the precision and applicability of this intervention strategy.

**Conclusion:**

Leucine‐enriched whey protein supplementation combined with resistance training is beneficial in addressing sarcopenia in older adults. This review consolidates the current understanding of this intervention, highlights its potential benefits and provides insights for future research and clinical applications in geriatric muscle health.

## 1 Introduction

Sarcopenia is characterised by the slow and progressive degradation of muscle mass, strength and functioning. Usually, sarcopenia prevails in older adults starting from age 50 years and above, and it slowly increases at a rate of 0.8% per year. Moreover, sarcopenia causes a rapid decrease in skeletal muscle mass, which is known as dynapenia and is increasing at a rapid rate of 2%–3% per year, and it is estimated to be 20% in older adults above the age of 65 and 50% in adults aged 80 years and above. It is also seen that in some diseases and conditions, such as liver cirrhosis, COPD and diabetes, the progression of sarcopenia occurs earlier and at a much faster rate. Many studies have highlighted that individuals with sarcopenia are most likely to develop conditions such as increased frailty, disability, improper muscle functioning and, in some cases, diabetes mellitus type 2. It also greatly increases the risk of falls in individuals and hinders their daily activities [1]. The SARC‐F screening tool was developed by Malmstrom and Morley (2013). This simple and rapid diagnostic test is widely used to diagnose sarcopenia. It has the following five components: these included muscle strength, walking assistance, rising from a chair, climbing stairs and falling. It has a score for every component, which ranges from 0 to 10, with 0 to 2 points. A score greater than 2 points is indicative of sarcopenia. Moreover, it is important to diagnose this condition rapidly and easily so that different interventions can be administered to the patient. These interventions included resistance training and protein and vitamin supplementations [2].

A meta‐analysis of the prevalence of sarcopenia in different parts of the world was carried out in which a total of 35 articles were selected. The prevalence was approximately 10% in both men and women aged > 60 years. It was also reported that the prevalence of sarcopenia was higher in non‐Asian individuals, with muscle mass of 19% in men and 20% in non‐Asian women and 10% in men and 11% in women who were Asians. It was also reported that many older adults have this condition [3]. A present‐day study was conducted on the US population, the National Health and Nutrition Examination Survey, which showed that muscle mass is the major predictor of mortality, especially in males and females over 55 and 65 years. Loss of muscle mass generally occurs with the progression of some chronic diseases, along with protein formation and degradation in the skeletal muscle tissues of humans. Another factor contributing to the loss of muscle mass is the anabolism of proteins at later ages, which can cause conditions such as sarcopenia. The literature suggests that individuals with sarcopenia need 2% more protein intake per meal than older individuals for proper protein synthesis and functioning. Furthermore, globally, approximately 40% of older individuals do not meet the approved daily protein intake, and about 10% of women do not meet the daily average requirements for protein, which leads to conditions such as sarcopenia [4].

A study was conducted in the city of Jhelum, province of Punjab, Pakistan, to identify the prevalence of sarcopenia in older adults. The study reported that out of the 36 participants who were screened from a sample of 222 people, 21 females (6.76%) and 15 males, which accounts for 6.76%, were deemed to have sarcopenia. Sixteen percent of participants had sarcopenia. Overall, a low prevalence of sarcopenia has been reported [5]. Early diagnosis and intervention play key roles in the prevention of sarcopenia. According to several studies, an intake of 1.0–1.2 g/kg of body weight dietary protein and 25–30 g of high‐quality protein per day per meal is recommended. Potential supplements that can prevent sarcopenia include protein supplementation using milk or cheese, essential amino acids, leucine and vitamin D, to increase muscle mass in older adults [6]. Sarcopenia includes an increase in inflammatory biomarkers, oxidative stress and a significant decrease in anabolic markers of muscle. Whey protein is considered a high‐quality dairy protein and is widely used. A total of 35 g of whey has been reported to improve sarcopenia biomarkers in significantly frail patients. Whey protein has been shown to improve the mTOR signalling pathway in older adults [7, 8].

Another study evaluated the effects of strength training and leucine‐enriched whey protein supplementation on muscle mass, strength and function in older adults with sarcopenia. This study was conducted in Japan and was a randomised controlled trial. The study included individuals aged 65 years and above who were randomly allocated to three groups. The groups received whey protein supplementation and resistance training; the second group included resistance training only, and the third group was given protein supplementation only. All the groups included 27 individuals in this study. The study was conducted for 24 weeks, and protein supplementation included 11 g of protein and 2.3 g of leucine. Appendicular muscle mass, handgrip strength, body mass index (BMI) and ideal body weight were measured at baseline and after the intervention. The results showed that appendicular muscle mass and handgrip strength significantly increased by the end of the intervention in the protein along with resistance training group (*p* < 0.01). After 24 weeks, appendicular muscle mass and handgrip strength increased in the resistance training along with the protein group compared to resistance training alone. The study concluded that resistance training and protein supplementation are beneficial in preventing and mitigating the effects of sarcopenia in older adults [9].

Sarcopenia significantly affects mobility, independence and overall quality of life in older adults, increasing their risk of falls, fractures and hospitalisation. Traditional treatments fail to fully combat this multifactorial disease. Recently, there has been interest in nutritional strategies, particularly leucine‐enriched whey protein, along with resistance training. However, despite the growing interest in this field, there is a lack of comprehensive reviews that emphasise the combined effects and underlying mechanisms. This article aims to address this gap in the literature to determine the efficacy and applicability of this intervention.

Leucine stimulates muscle protein synthesis via the mTOR pathway, whereas resistance training enhances muscle strength and adaptation. Together, they work synergistically to counteract muscle loss in older adults. Evidence has shown that it improves lean mass, strength and physical performance.

## 2 Methods and Materials

### 2.1 Search Strategy

An extensive search strategy was used to retrieve relevant data and articles from primary databases, including Google Scholar, Wiley, PubMed, Scopus and NCBI. The main focus in finding the appropriate data and information was on the titles and abstracts of the articles. Keywords such as ‘sarcopenia, ‘mTOR,” ‘older adults,’ ‘sarcopenic adults,’ ‘leucine,’ ‘whey protein,’ ‘whey protein isolate,’ ‘leucine‐enriched whey protein,’ ‘resistance training,’ ‘home‐workout,’ ‘elderly,’ ‘sarcopenic obesity’ and ‘muscle mass’ were used to find appropriate and relevant articles. This article systematically reviews randomised controlled trials, longitudinal studies and meta‐analyses published between 2011 and 2023.

### 2.2 Inclusion Criteria

This review specifically focused on randomised controlled trials of leucine‐enriched whey protein in combination with resistance training to mitigate the sarcopenic effect in older adults. It also includes other chronic diseases closely related to sarcopenia that intensify muscle loss and cause chronic sarcopenia. Moreover, the effects of leucine and whey protein on the mTOR signalling pathway, which plays a key role in maintaining muscle mass, were observed. Furthermore, studies that included resistance or strength training for older adults have been conducted.

## 3 Part I: Sarcopenia and Disease Relation

### 3.1 Sarcopenic Cancer Patients

Malnutrition is highly prevalent in individuals with cancer and has a huge negative impact on the tolerance of cancer treatments, clinical outcomes and the rate of survival. Therefore, it is highly recommended that early nutritional support be provided along with early nutritional screening [10]. Many different oral nutritional supplements are currently recommended for cancer patients. However, the literature lacks evidence to explain the effects of leucine‐enriched oral nutritional supplements in cancer patients. This study aimed to evaluate the effects of standard hypercaloric oral nutrition supplements with whey protein–based oral nutrition versus leucine‐enriched oral nutrition in cancer patients and was a 12‐week clinically controlled trial. The control group was administered a whey protein–based nutritional supplement, and the intervention group received a leucine‐enriched oral nutritional supplement. A total of 46 patients took part in the study, and anthropometric measurements were taken, along with ultrasound of the muscles, quadriceps and abdominal areas and biochemical profile. All patients were administered vitamin D supplements throughout the study period.

**FIGURE 1:**
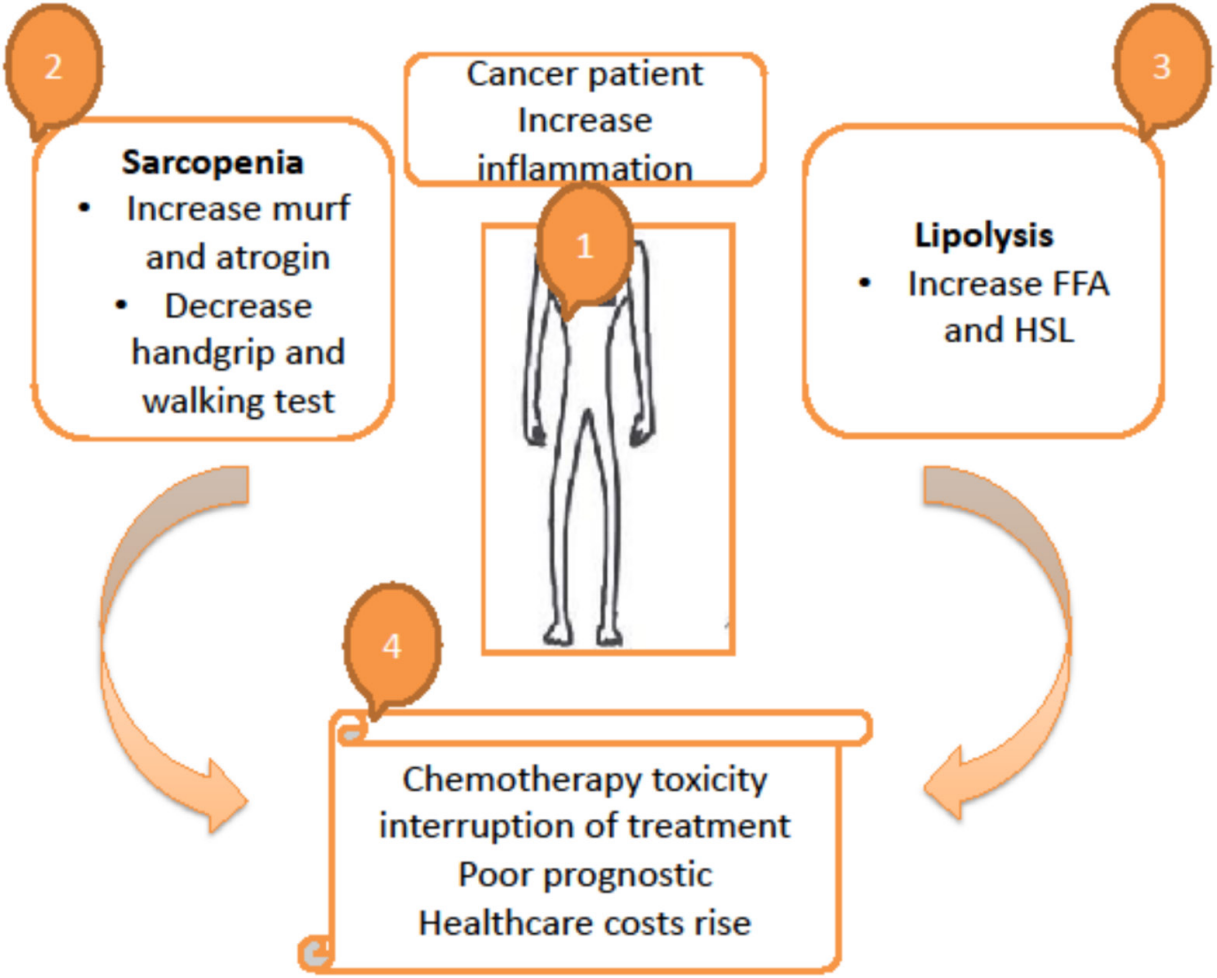
Sarcopenia in cancer patients.

The results indicated that all nutritional biomarkers, including anthropometric measurements, bioimpedance analysis, ultrasound and biochemical profiles, were fairly stable for all patients, and the extracellular mass increased in the leucine‐enriched formula intervention participants. Muscle function also increased in both groups. Pre‐albumin and transferrin levels increased in the control group (*p* < 0.05). Self‐reported quality of life increased in both groups. The study concluded that whey protein–based oral supplementation for cancer is beneficial for muscle functionality, along with quality of life and maintenance of body composition in cancer patients when paired with vitamin D supplementation. Overall benefits were observed with the intervention, which included leucine‐enriched oral supplementation (Figure 1) [11].

This study aimed to investigate the effects of whey protein on the onset of this condition, especially in patients with colorectal cancer undergoing 5‐fluorouracil–based chemotherapy. The patients were randomly divided into the whey protein and placebo groups. The patients were blinded and randomised. Their nutritional status and physical measurements were taken before the start of the chemotherapy and then at the 3‐ and 6‐month marks, which also included tools such as the Mini Nutritional Assessment (MNA) and Malnutrition Universal Screening Tool. Forty‐seven patients were included in this preliminary study. During chemotherapy, 33 patients were evaluated, and their anthropometric measurements, including lean body mass, increased from 68.5% to 71.2% and 68.7% to 66.3% in the whey protein and placebo groups, respectively. Moreover, the results indicated that sarcopenia percentage decreased from 4% to 54% in the whey protein group. The study concluded that whey protein supplementation could be beneficial in preventing the onset of sarcopenia, improving nutritional status and reducing toxicity in colorectal cancer patients undergoing chemotherapy [12, 13].

### 3.2 Mechanism I: mTOR Signalling Pathway in Older Adults

An anabolic resistance response in the presence of activation of the mTOR signalling pathway can characterise sarcopenia. A shift of fibres in the muscle composition towards the slow twitch of the fibres accompanied by reduced function of the glucose transporter GLUT‐4 is seen in older individuals, which is further accompanied by reduced functioning of the muscle to utilise glucose upon insulin stimulation. All of these are associated with certain metabolic disorders, such as obesity and diabetes mellitus, in which insulin stimulation is altered. Skeletal muscles can be considered endocrine organs that help release different molecules and control the metabolism of the human body. In patients suffering from liver cirrhosis, it can be seen that there are alterations and changes in the mTOR signalling pathway, which is an indicator of sarcopenia [14]. A possible biomarker of sarcopenia is the reduction in the circulation of IGF‐1 levels in the body, which is often related to sarcopenia and is an indicator of sarcopenia in older adults [15]. Many studies have shown that as an individual ages, the levels of IGF‐1 subsequently decrease, whereas the levels of IGF‐1 receptor cease in older adults. Hence, activation of the signalling pathway is greatly accelerated in individuals with sarcopenia [16].

**FIGURE 2:**
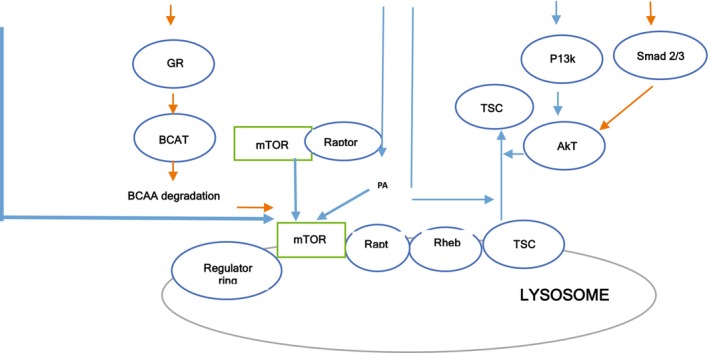
mTOR signalling pathway. Following is the summary of the mTOR signalling pathway in skeletal muscle. This pathway is affected by multiple factors, and it is activated by insulin, mechanical stimulation and amino acids, which are represented by blue line. It is inhibited by glucocorticoids and myostatin, which is represented by orange lines [18].

Many studies have explained the benefits of physical exercise in alleviating sarcopenia symptoms [17, 18]. An increase in protein loss, degradation, deterioration and disuse of protein synthesis causes loss of skeletal muscle mass, muscle deterioration and muscle atrophy, which can be triggered by aging or any other chronic disease that ultimately causes sarcopenia in older individuals. mTOR causes muscle hypertrophy, and when paired with myostatin and glucocorticoids, muscle deterioration occurs as they send muscle atrophy signals to the body (Figure 2) [19].

The mTOR signalling pathway is activated by numerous nutrient‐sensing signals, including different types of amino acids and energy sources. mTOR is a nutrient response signalling pathway that incorporates different growth factors, including amino acid availability, energy and stress, which help regulate cell growth and proliferation. mTOR comprises two types of complexes: mTOR signalling pathway complex 1 and mTOR signalling pathway complex 2. Complex 1 generally controls the synthesis of proteins and fats and caters to human stress responses that lead to autophagy. Complex 2 regulates the insulin response and signalling and includes proteins such as rapamycin, which has an anti‐tumour effect in the body. Both complexes contain similar mTOR proteins and domains that contain mTOR interacting proteins [20].

### 3.3 Mechanism II: Leucine and mTOR Signalling Pathway

Many studies have shown that ingesting leucine‐enriched amino acid interventional products enhances the activation of rapamycin in the mTOR signalling pathway in the human body and helps synthesize the protein in regulating skeletal muscle mass in humans. Moreover, when leucine‐enriched amino acid supplementation was performed with resistance training, protein turnover was greatly increased in the muscles. Adding leucine to older individuals' meals may greatly stimulate protein synthesis in the muscles of older individuals with sarcopenia. It is still unknown how the human body senses a rise in leucine‐enriched amino acid supplementation levels to activate the mTOR signalling pathway. However, recent studies have reported that hVps34 kinase and MAP 43 k (also known as GLK) are threonine and serine kinases that may be actively involved. Leucine‐enriched amino acid supplementation coupled with resistance training may be beneficial in many conditions that involve muscle degradation or muscle wasting, including sarcopenia, AIDS, kidney failure and sepsis [21].

Another study has been conducted on the use of protein energy supplementation to overcome muscle mass loss. In a 2‐week study, older adults were given leucine supplementation measuring 4 g per meal over three meals per day. Metabolic research was conducted on days 1 and 15 post‐intervention, and leucine supplementation was not administered. A muscle biopsy and blood profile analysis were performed for each participant using l‐phenylalanine. Markers of nutrient signalling, including mTOR signalling pathways, were measured along with the fractional synthesis rate of muscles pre‐ and post‐intervention. The results indicated that, after the 2‐week supplementation with leucine, the fractional synthesis rate increased along with mTOR activation. However, there was no significant difference in fat mass. The study concluded that leucine supplementation in older adults benefits muscle protein synthesis and mTOR signalling pathway activation [22, 23]. Leucine supplementation has proven to be beneficial for the activation and stimulation of mRNA translation, which is important for the activation of the mTOR signalling pathway in older adults, as well as for promoting its phosphorylation by activating the eukaryotic initiation factor, which is a binding protein along with ribosomal proteins such as S6 kinase 1 and ribosomal protein S6, which further increases protein turnover in the muscles and prevents muscle atrophy. Leucine also positively affects mRNA translation, which is important for activating the mTOR pathway [24].

### 3.4 Mechanism III: Sirtuin Pathway and Sarcopenia Association

SIRT1 plays an important role in the development of sarcopenia in older adults because of its key roles in regulating protein homeostasis, apoptosis, mitochondrial dysfunction, insulin resistance and autophagy in the skeletal muscles [25]. The sirtuin pathway is closely related to aging and frailty, which are the key factors in sarcopenia. It consists of seven isoforms, called nicotinamide adenine dinucleotide (NAD^+^) proteins. It also helps mediate the effects of a caloric‐restricted diet, which is the only solution to slow aging [26]. Another study validated that SIRT1 and SIRT3 help slow the aging process in older individuals [27]. Moreover, a similar study highlighted that SIRT1 and SIRT3 serum levels significantly decrease with increasing frailty in sarcopenic individuals [28]. Moreover, PGC1α deacetylation by SIRT1 stabilises the mitochondria in skeletal muscles. Oxidative stress is one of the key aspects of sarcopenia and is significantly higher in frail individuals due to increased levels of glucose‐6‐phosphate dehydrogenase.

**FIGURE 3:**
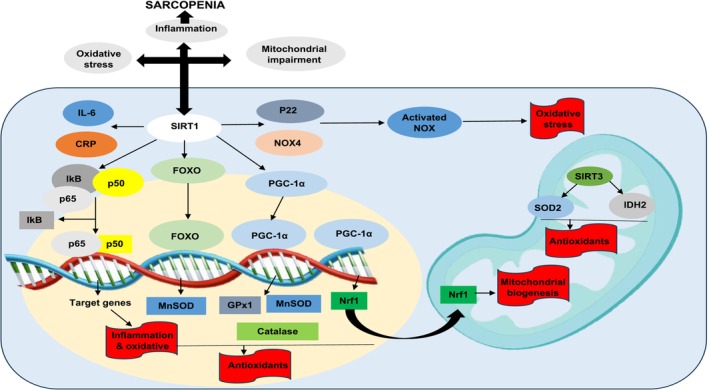
Sirtuin pathway and pathophysiology of sarcopenia. IL‐6 = interleukin‐6, SIRT1 = sirtuin 1, CRP = C‐reactive protein, FOXO = fork head box transcription, NOX4 = NADPH oxidase 4, IkB = IkappaB kinase, MnSOD = superoxide dismutase 2, PGC‐1α = peroxisome proliferator‐activated receptor gamma coactivator, IDH2 = isocitrate dehydrogenase 2, Nrf1 = nuclear respiratory factor, Gpx1 = glutathione peroxidase 1.

Furthermore, it was observed that inflammation increased in patients who were frail and had sarcopenia due to the higher levels of interleukin‐6, which is another hallmark of sarcopenia and eventually leads to sarcopenia. This depends on SIRT1 deacetylation and activation of the PGC1α, which plays a major role in mitochondrial biogenesis [26]. Currently, only some interventions are available for down‐regulating the SIRT1 pathway, including polyphenolic compounds, exercise and a calorie‐restricted diet [25]. Further studies are warranted to determine the full potential of SIRT1 (Figure 3).

## 4 Leucine as Branched‐Chain Amino Acid

Leucine, isoleucine and valine are branched‐chain amino acids (BCAAs) that contain a functional R group and are considered essential amino acids. Leucine is an important activator of the mTOR signalling pathway and promotes muscle protein synthesis. The levels of BCAAs increase after exercise when they are consumed pre‐workout [29]. A recent study showed that supplementation with leucine 10 g per day along with resistance exercise for 13 weeks helped increase the gait speed of sarcopenic individuals [1].

This study investigated the impact of BCAAs leucine, isoleucine and valine on mortality in malnourished, polymorbid inpatients using data from the EFFORT trial. These essential amino acids support protein synthesis, muscle recovery and glucose regulation. Among 238 patients with metabolite data, low leucine levels were linked to more than double the risk of 180‐day all‐cause mortality (adjusted HR, 2.20), while low isoleucine and valine levels were also significantly associated with higher mortality risks (HR, 1.56 and 1.69, respectively). However, nutritional support did not significantly reduce the mortality of patients with low BCAA levels. These findings suggest that BCAA depletion is a strong predictor of poor outcomes; however, standard nutritional interventions may not adequately address this risk, warranting further research into targeted strategies [30].

## 5 Role of Resistance Training in Sarcopenia

**FIGURE 4:**
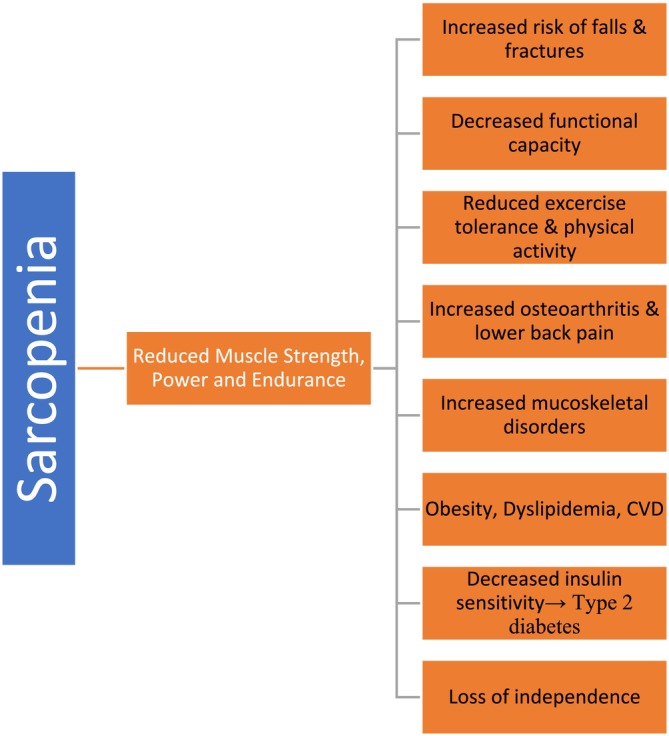
Role of resistance training in sarcopenia.

The literature explains the beneficial role of resistance training in preventing the onset and symptoms of sarcopenia. Different American societies, including the National Strength and Conditioning Association and the American College of Sports Medicine, have published different guidelines that include how to maintain and boost muscle strength with a combination of types of exercises, intensity and volume, along with frequency best suited for muscle mass improvement and gain. The type of resistance training required and its effectiveness depend on the person and their level of fitness and exercise history for a better outcome. The level of resistance training intensity required to increase skeletal muscle mass and improve muscle function, along with muscle strength, is 65% or above of one‐repetition maximum (1RM) [31]. Moreover, resistance training below 65% to 70% of that of 1RM caused no significant improvement in muscle function in young and older adults, respectively. High‐load resistance training is considered a good measure for preventing sarcopenia. Many studies have shown that resistance training is effective in protein synthesis in muscles and increases skeletal muscle mass in both young and older adults (Figure 4) [32, 33].

**FIGURE 5:**
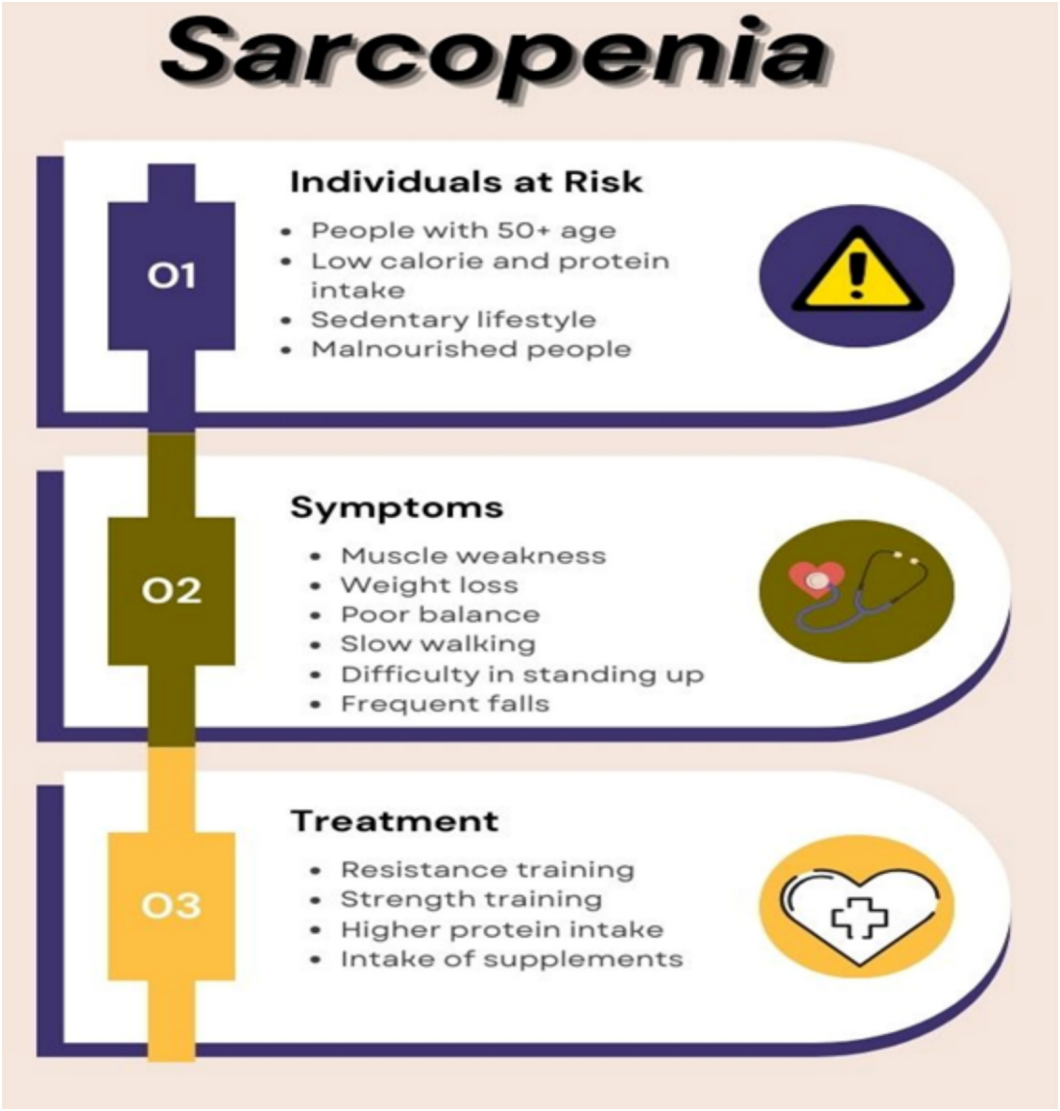
Individuals at risk, symptoms and treatment of sarcopenia.

A study was carried out in which the effect of continuous resistance training was observed in hospitalised older adults with sarcopenia. The participants were from a nursing care facility where they participated in controlled trial research in which a programme was developed for them to have resistance training exercises and balance training every 2 days per week for the next 6 months. They were divided into two groups containing 21 participants; their mean ages were 85.9 ± 7.5 years, and the patients were measured according to the European Working Group on Sarcopenia for older adults. At baseline, the prevalence of sarcopenia was approximately 35%. The prevalence increased significantly in the control group post‐intervention, from almost 42.9% to 52.4%. The results showed that the resistance training group had an increase in hand grip strength compared to the control group, and the exercise group showed a decrease in BMI compared to the control group. The study concluded that resistance training positively affected older adults' BMI and hand grip strength in nursing facilities. Individuals with sarcopenia can also benefit from resistance training and minimise symptoms (Figure 5) [34, 35].

Sarcopenia is becoming a major public health problem worldwide, and it is known to be the loss or degradation of muscle mass, function and strength [36]. This study aimed to determine the effects of high‐intensity resistance training, which is a time‐ and cost‐effective training programme for older adults with sarcopenia and osteosarcopenia. A total of 43 male individuals were selected for the study from northern Bavaria, Germany, and were randomly allocated to two groups: a high‐intensity resistance training group and an inactive control group. Both groups were given dietary protein supplementation, and the high‐intensity resistance training group received 1.5 g per kg per day. In contrast, the control group was given 1.2 g per kg daily and vitamin D supplementation up to 800 IE/day. The high‐intensity resistance group was meticulously supervised, and they were given a single set of training sessions using machines. The training sessions were conducted 2 days per week for 28 weeks. The sarcopenia *Z* score, skeletal muscle mass index, hand grip strength and gait velocity were also measured in both groups. The results showed a significant improvement in the exercise group's *Z* score compared to that of the control group, which had a worse *Z* score than the baseline.

**FIGURE 6:**
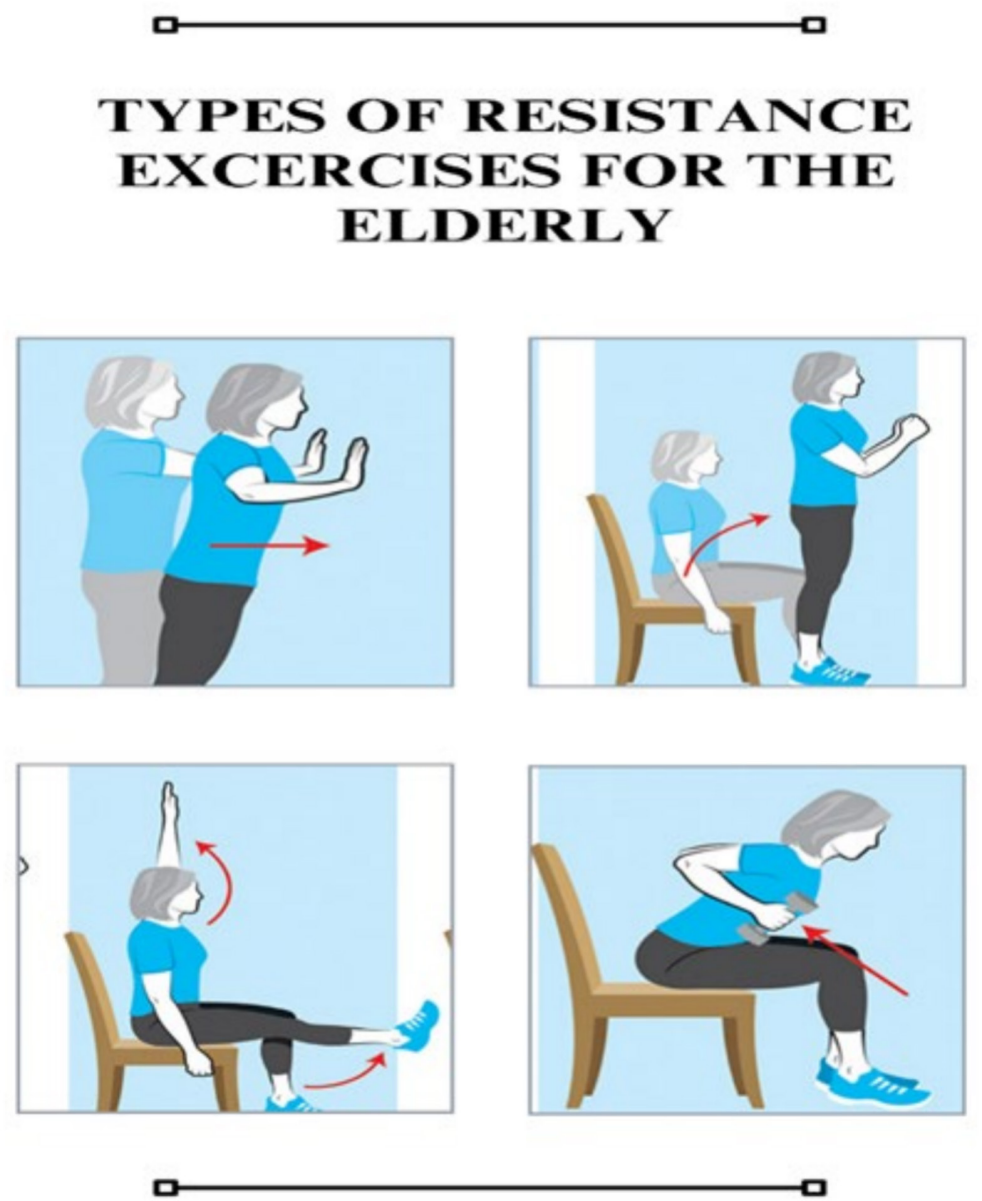
Types of resistance exercises for older adults to do at home.

Moreover, the skeletal muscle mass index and hand grip strength also significantly increased in the resistance training group [37]. Neither group had negative effects on dietary protein or vitamin D supplementation. The study concluded that high‐intensity resistance training is an effective measure for sarcopenic older adults, as it is time‐ and cost‐efficient and provides desirable results in combating sarcopenia [38, 39]. When discussing sarcopenia, the literature suggests that resistance training is an important part of, as it is effective in increasing muscle strength and stimulating muscle hypertrophy. Both the elderly and frail individuals benefit from regular resistance training exercises as they induce skeletal muscle hypertrophy. The only way to achieve muscle hypertrophy is to achieve a good balance between protein breakdown and synthesis in the muscles. In simpler words, to achieve beneficial results, there should be harmony between protein intake and breakdown. The literature also indicates that resistance training increases muscle protein synthesis and MCH‐specific proteins, which are mostly found in young populations. Resistance training also increases the rate of muscle fractional synthesis in frail sarcopenic individuals (Figure 6) [40].

## 6 Whey Protein as an Amino Acid Source

Milk and its components are widely known as functional foods that directly influence human health. Milk has two major protein types: whey and casein. With advancements in the food processing industry, many different products containing whey protein for human consumption have been obtained with the help of ultrafiltration and microfiltration processing [41]. There are various types of whey protein, the most famous of which are whey protein concentrate, which is 80%–95% protein with or without lactose; whey protein isolates, which is 90%–95% protein and normally has 0%–1% carbohydrate; and whey protein hydrolysate, which has the same protein content as the isolate and results in fewer allergies in humans upon consumption. Liquid whey comprises approximately 20% of the total protein content in bovine milk. Whey protein can be considered a mixture of different amino acids, as it mainly contains approximately 26% BCAA, including l‐lysine, l‐arginine and l‐glutamine. It also contains sulphur‐containing amino acids such as cysteine and taurine [42].

**FIGURE 7:**
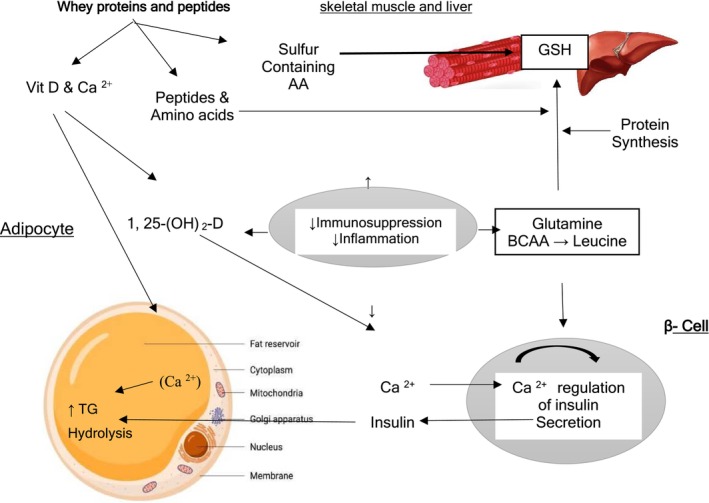
Different mechanisms involving whey protein and leucine as immune nutrients. Whey protein induces protein breakdown and synthesis in muscles, influences lipid metabolism and affects the antioxidant system. Ca^2+^ = calcium, 1,25‐(OH)_2_ = 1,25‐hydroxycholecalciferol, [Ca^2+^] = intracellular calcium concentration, TGs = triacylglycerol [35].

Whey protein is a rich source of bioactive components, including immunoglobulins such as albumin and lactoferrin, as well as essential nutrients such as calcium and vitamin D. Lactoferrin exhibits potent antimicrobial properties, whereas other globulins contribute to antibacterial and antiviral effects. In the context of chronic inflammatory conditions and elevated adiposity, whey protein, especially when combined with calcitropic hormones such as parathyroid hormone and 1,25‐dihydroxycholecalciferol, has been shown to significantly reduce inflammation. Additionally, studies have highlighted the role of whey proteins in regulating glucose homeostasis and inhibiting adipogenesis, further supporting their anti‐inflammatory potential. [43]. Beyond metabolic benefits, whey protein also mitigates oxidative stress induced by resistance exercise and helps preserve the redox balance within immune cells, thereby enhancing the overall immune function (Figure 7) [42, 44].

## 7 Leucine‐Enriched Whey Protein for Sarcopenia

It has been observed that resistance training is an important factor in counteracting the condition sarcopenia, which is an age‐related loss of muscle mass. This study aimed to scrutinise the outcome of resistance exercises coupled with leucine‐enriched whey protein post‐exercise in older adults who were frail and post‐hospitalised, as well as the effect of this intervention on myokine levels. This study included 41 individuals aged > 65 years with sarcopenia. The study was conducted over 12 weeks using a resistance training programme. This was a randomised placebo‐controlled trial. Sarcopenia screening, frailty testing and fasting blood glucose levels were measured at the start of the study and after the 12‐week period. The placebo group was given no intervention, but still participated in the resistance training programme. They were asked to maintain good dietary protein intake. The results of this study showed that both groups had improved physical performance and frailty status. However, in the intervention group, appendicular skeletal muscle mass improved slightly compared to the placebo group [45].

Sarcopenia can worsen in older adults if a lack of exercise persists, as it is important not to prolong the state of inactivity in older adults with sarcopenia. The objective of this study was to investigate the outcomes of leucine‐enriched whey protein in addition to a well‐developed resistance training programme of 12 weeks. Twenty‐eight volunteers were included in this resistance training programme, which was performed every 2 days per week. The control group received the intervention and resistance training, whereas the placebo group received only resistance training. Physical function, frailty level, body mass, short physical performance battery test and MNA were conducted. The results showed no significant improvement in individuals with 2 days of resistance training per week for 12 weeks [46]. Another test that can be used to assess older adults' physical activity is the senior fitness test, previously known as Fullerton's fitness test. The test has six components. The first component is the lower body strength test, which is a 30‐s chair stand test. In this case, the individual has to perform a set number of stands from the chair in 30 s with their hands folded on the chest. The second part tests the upper body strength, including the arm curl test, in which the patient's number of bicep curls is measured at 5 lb for women and 8 lb for men. Then, there was a 6‐min walk test across 45 m. The fourth test was the chair sit and stand test, which was followed by the flexibility back test, which included the patient extending one arm over the shoulder towards the back and the second arm from the downside and measuring the distance between them. The last stand is the walking test, in which the individual is asked to walk 8 ft and return to the chair (Table 1) [47].

**TABLE 1:** Randomised controlled trials in older adults with whey protein and leucine intervention.

References	Study type	Study period	Participants	Age	Intervention and dosage	Frequency of supplementation	Outcome
Cruzat et al. [42]	Randomised double‐blind placebo‐controlled trial	6 weeks	CG *n* = 12 IG *n* = 12 (All males)	71 ± 4 years	3 g Leucine 20 g Whey protein 800 g Vitamin D	Once daily before breakfast	↑ Postprandial FSR and appendicular lean mass in IG
Ascenzi et al. [10] Johnston et al. [40]	Randomised double‐blind placebo‐controlled trial	4 weeks	CG *n* = 30 IG *n* = 31 (Males and females)	85.0 ± 7.4 years	15 g WP 5 g EAA 550 mg Calcium 30 mg/day Zinc	Once daily	↑ Serum IGF‐I Bone resorption ↑ Daily activities
Coker et al. [17]	Randomised controlled trial	8 weeks	CG *n* = 6 IG *n* = 6 (Males and females)	65–80 years	800 kcal/day EAAMR 400 kcal/day CMR	Once daily	Adipose tissue in EAAMR In 7% body weight in both groups ↑ FSR
Pennings et al. [31]	Randomised controlled trial	1‐Day trial and testing	IG 1 *n* = 11 IG 2 *n* = 11 IG 3 *n* = 11 (All males)	73 ± 2 years	10, 20 and 35 g of phenylalanine‐labelled WP	Once daily	35 g WP ↑ Amino acid absorption ↑ Muscle protein synthesis
Borack et al. [12]	Double‐blind randomised controlled trial	1‐Day trial and testing	WPI + RT *n* = 10 PBSD + RT *n* = 9 (All males)	55–75 years	30 g WPI PBSD = 25% soy, 25% WP and 50% casein	Once post exercise	↑ Hyperamino‐acidemia, mTORC1 and muscle protein synthesis
Reitelseder et al. [35]	Randomised controlled trial	1‐Day trial and testing	WPH + RT *n* = 10 Caseinate + RT *n* = 9 CHO + RT *n* = 8 (All males)	69 ± 1 years	0.45 g/lean body weight of WPH, CAS, CHO drink	Once in fed state	↑ Myofibrillar protein synthesis
Kramer et al. [23]	Randomised double‐blind placebo‐controlled trial	1‐Day trial and testing	CG (healthy) = 15 IG (sarcopenic) = 15 (All males)	75 ± 1.4 years	Leucine‐enriched WP drink 21 g Protein 9 g CHO 3 g Fat 3 g Leucine	Once postprandial	↑ Muscle protein synthesis in both groups

Abbreviations: CAS = caseinate, CG = control group, CHO = carbohydrate, CMR = competitive meal replacement, EAA = essential amino acid, EAAMR = essential amino acid meal replacement, FSR = fractional synthesis rate, IG = intervention group, PBD = protein blend soy drink, RT = resistance training, WP = whey protein, WPH = whey protein hydrolysate, WPI = whey protein isolate.

This study investigated differences in the digestion and absorption of different amino acid proteins, their composition and their impact on postprandial muscle protein growth. This study aimed to compare whey protein, casein protein and casein hydrolysate digestion and absorption in older men with sarcopenia. Forty‐eight participants were above 70 years of age and were randomly assigned to different protein groups with 20 g of each protein group. Protein ingestion was coupled with the intravenous administration of l‐phenylalanine to assess the absorption and digestion of proteins. The results showed that ESR values were the highest after whey protein intake. The study concluded that whey protein stimulates muscle protein growth most after ingestion in older men because of its higher leucine content [48].

This study aimed to determine the effects of consuming pure isolated micellar casein or ingesting it at rest and after a resistance training session. Myofibrillar proteins are responsible for the changes in skeletal muscle mass after hard exercise. Healthy men above 71 years of age participated in this study, with a mean BMI of 26.4 kg/m^2.^ They were divided into two groups of seven participants each, and both groups were administered a dose of l‐phenylalanine to calculate myofibrillar protein synthesis. One group was administered 20 g of casein, and the other group received 20 g of whey protein after performing leg resistance exercises, and the results were calculated after 4 h of exercise. First, it was observed that after 60 min of ingestion of whey protein, leucine and amino acid concentrations were the highest in the blood. Moreover, it was seen that when the leg was at rest, the myofibrillar protein synthesis was 65% higher in patients who ingested whey protein than in the casein group. Similarly, after exercise, the MPS value of the whey group was greater. Therefore, this study concluded that whey protein stimulates myofibrillar protein synthesis more than casein protein [41].

The main purpose of this study was to compare the pre– and post–muscle protein synthesis rates in healthy individuals and individuals with sarcopenia. Two male groups participated in this study, with 15 participants in each group. The mean age of the healthy group was 69 years, and that of the men with sarcopenia was 81 years. Both groups consumed leucine‐enriched whey protein supplement. The composition was 21 g of proteins, 9 g of carbohydrates and 3 g of fat. Stable isotope and recurrent blood and muscle mass sampling were used to assess prepostprandial and postprandial muscle protein synthesis rates. Sarcopenic males were identified according to international criteria, which included gait speed, muscle mass and handgrip strength. After the results were calculated and compared, the study concluded that basal and postprandial muscle protein synthesis was in both the healthy group and sarcopenic males. So, it is safe to say that 21 g of leucine‐enriched whey protein supplementation successfully raised the rates of muscle protein synthesis in older men of both groups (Table 2) [23].

**TABLE 2:** Leucine‐enriched whey protein and its outcomes.

Study	Study type	Study period	Participants	Intervention and dosage	Frequency of supplementation	Outcome
Bauer et al. [49]	Randomised controlled trial, double‐blind study	13 weeks	CG *n* = 196 IG *n* = 184 with RT	6 g Leucine 40 g Whey protein 18 g CHO 6 g Fat 1600 g Vitamin D	Twice daily	Increase in appendicular muscle mass, chair stand test and muscle strength
Kou et al. [50]	Randomised controlled trial	12 weeks	CG *n* = 12 IG *n* = 12 with RT	20 g whey protein 2.8 g leucine	Once daily	Increase in post‐exercise muscle protein synthesis
Nabuco et al. [7]	Randomised controlled trial	12 weeks	CG *n* = 26 with RT IG *n* = 26 with RT	35 g Whey protein	Once daily	Increase in appendicular lean soft tissue and decrease in trunk fat mass in the intervention group
Mori and Tokuda [9]	Randomised controlled trial	24 weeks	RT + PRO *n* = 27 RT *n* = 27 PRO *n* = 27	11 g Whey protein 2.3 g Leucine	Twice per week	ASMI and HGS increased in RT + PRO

Abbreviations: ASMI = appendicular skeletal muscle index, CG = control group, IG = intervention group, PRO = protein, RT = resistance training.

This randomised controlled study aimed to assess the effectiveness of combining detraining with leucine‐enriched whey protein supplementation and resistance exercise on muscle mass and strength in older adults diagnosed with sarcopenia, a condition characterised by age‐related loss of muscle mass. Conducted in a community setting in Japan, the study included participants aged 65 years and older who were screened for sarcopenia and randomly assigned to one of three groups: (1) whey protein supplementation combined with resistance training (*n* = 27), (2) resistance training only (*n* = 27) and (3) whey protein supplementation only (*n* = 27). The intervention lasted 24 weeks, followed by a 24‐week detraining period to evaluate the sustainability of the outcomes. The protein supplement consisted of 11 and 2.3 g, respectively. Resistance training and protein supplementation were administered twice weekly, and appendicular skeletal muscle mass and handgrip strength were calculated at the end of the intervention period and at the 12‐ and 24‐week marks of the training programme. The results of both groups were compared, and it was observed that there was a slight increase in the appendicular skeletal mass and handgrip strength in the group with protein and resistance training combined, but there was no notable difference between the groups. However, resistance training was unsuccessful after the detraining period, and the combination of proteins with resistance training showed promising results in the long term [49, 50].

The key aim of this study was to examine the effects of leucine administration, which is an essential amino acid, in individuals with sarcopenia aged > 65 years and whether the administration of leucine can improve muscle mass, muscle strength and functional and respiratory muscle performance of individuals. Fifty participants were selected for this double‐blind randomised controlled trial. The study was conducted over 13 weeks, with the control group given 6 g of leucine per day and the placebo group given 6 g of lactose per day. This study evaluated primary and secondary outcomes. The primary outcomes included sarcopenia and respiratory muscle function, and the secondary outcomes included cognitive function and nutritional assessment. The results showed that the leucine administration increased the walking time and lean mass index. In addition, respiratory function increased in the leucine group (*p* = 0.026) compared to the placebo group. Therefore, leucine can improve some aspects of sarcopenia [4].

## 8 Conclusion

In conclusion, the integration of leucine‐enriched whey protein with resistance training offers a clinically relevant and evidence‐based strategy for combating sarcopenia in older adults. Leucine plays a critical role in stimulating muscle protein synthesis and reducing muscle breakdown, thereby counteracting age‐related anabolic resistance. When combined with resistance exercises, this nutritional intervention has been shown to significantly improve muscle mass, strength and physical function, thereby enhancing mobility and supporting independence in the aging population. Although existing studies vary in design, dosing and participant characteristics, the overall findings consistently highlight the benefits of this approach. To effectively translate these findings into clinical practice, future research should aim to refine dosing strategies, evaluate long‐term outcomes and personalise interventions according to individual patient needs, ultimately facilitating broader implementation and promoting healthy ageing through targeted nutrition and exercise.

## Disclosure

The authors have nothing to report.

## Ethics Statement

This study did not involve humans or animals.

## Consent

This study did not involve humans.

## Conflicts of Interest

The authors declare no conflicts of interest.

## Data Availability

The data supporting the findings of this study are available from the corresponding author upon reasonable request.
